# Identification of Polyketide Inhibitors Targeting 3-Dehydroquinate Dehydratase in the Shikimate Pathway of *Enterococcus faecalis*

**DOI:** 10.1371/journal.pone.0103598

**Published:** 2014-07-29

**Authors:** Vivian Wing Ngar Cheung, Bo Xue, Maria Hernandez-Valladares, Maybelle Kho Go, Alvin Tung, Adeleke H. Aguda, Robert C. Robinson, Wen Shan Yew

**Affiliations:** 1 Department of Biochemistry, Yong Loo Lin School of Medicine, National University of Singapore, Singapore, Singapore; 2 Institute of Molecular and Cell Biology, Singapore, Singapore; 3 Synthetic Biology Research Consortium, National University of Singapore, Singapore, Singapore; University Hospital of the Albert-Ludwigs-University Freiburg, Germany

## Abstract

Due to the emergence of resistance toward current antibiotics, there is a pressing need to develop the next generation of antibiotics as therapeutics against infectious and opportunistic diseases of microbial origins. The shikimate pathway is exclusive to microbes, plants and fungi, and hence is an attractive and logical target for development of antimicrobial therapeutics. The Gram-positive commensal microbe, *Enterococcus faecalis*, is a major human pathogen associated with nosocomial infections and resistance to vancomycin, the “drug of last resort”. Here, we report the identification of several polyketide-based inhibitors against the *E. faecalis* shikimate pathway enzyme, 3-dehydroquinate dehydratase (DHQase). In particular, marein, a flavonoid polyketide, both inhibited DHQase and retarded the growth of *Enterococcus faecalis*. The purification, crystallization and structural resolution of recombinant DHQase from *E. faecalis* (at 2.2 Å resolution) are also reported. This study provides a route in the development of polyketide-based antimicrobial inhibitors targeting the shikimate pathway of the human pathogen *E. faecalis*.

## Introduction

The shikimate pathway comprises seven catalytic steps that convert the metabolic substrates D-erythrose 4-phosphate and phosphoenolpyruvate to chorismate in all microorganisms, including common enteric bacteria such as *Escherichia coli* and pathogenic microbes such as the vancomycin-resistant *Enterococci* (VRE) [1]. Chorismate is a necessary precursor in the biosynthesis of folate, aromatic amino acids and ubiquinone. The pathway is exclusive and crucial for microorganisms, plants and fungi.

The recent emergence of multi-drug resistant pathogenic microbes demonstrates a pressing need to develop new antibiotics. The absence of the shikimate pathway in humans presents an attractive target in the development of antimicrobials. Numerous studies have attempted to target enzymes in the shikimate pathway [2]. Currently, N-phosphomethylglycine is the only commercially available compound that targets one of the enzymes in the pathway; it targets 5-enolpyruvate shikimate-3-phosphate synthase [3], [4], [5].

**Figure 1:**
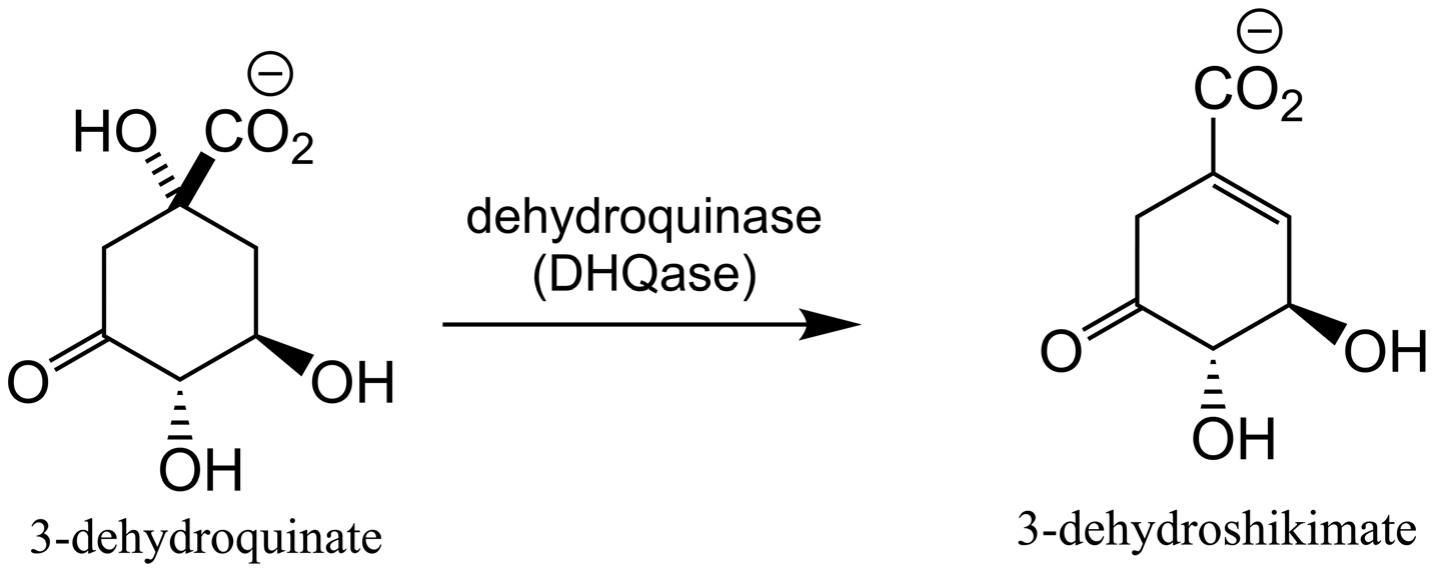
The DHQase reaction.

3-Dehydroquinate dehydratase (DHQase) is the third enzyme in the shikimate pathway. DHQase catalyzes the dehydration of 3-dehydroquinate to 3-dehydroshikimate (Figure 1). There are two types of DHQase: type I enzymes catalyze a Schiff base mechanism using a catalytic lysine residue; type II DHQase catalyze the dehydration reaction *via* an enolate intermediate. DHQase from *Enterococcus faecalis* is a type I enzyme. Other organisms that have type I DHQases include *Eschericia coli* [6], *Salmonella typhi* [7] and *Shigella dysenteriae* [8].

**Figure 2:**
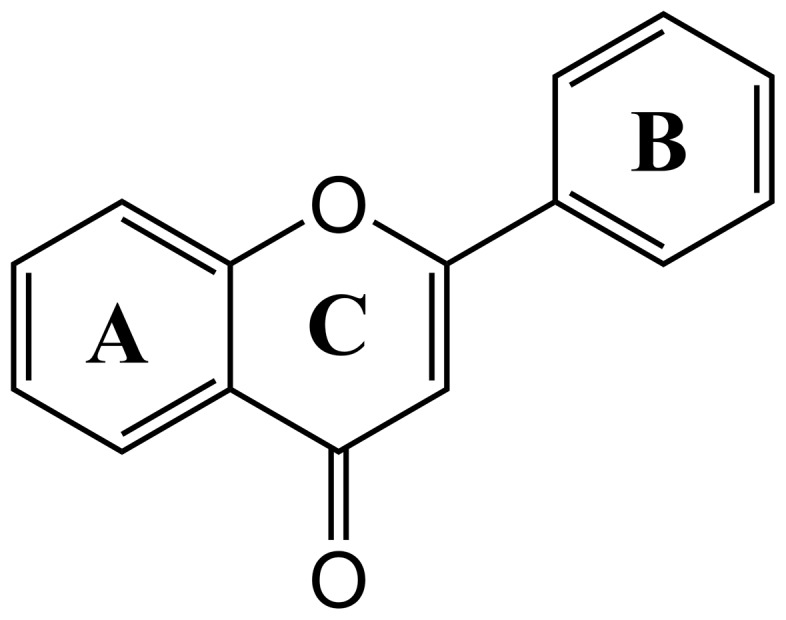
Backbone structure of flavones. Two benzene rings (A and B) linked through a heterocyclic pyrane ring (C).

Flavonoids are produced by plants as secondary metabolites, and include polyketide-based natural compounds. Polyketides are a large class of biomolecules that are naturally produced by bacteria, fungi and plants, and include many clinically important biomolecules with anti-cancer, anti-microbial, anti-oxidant and anti-inflammatory activities. Currently, there are about 3000 flavonoids extracted and characterized from plants [9]. The main function of these compounds is to provide colors attractive to plant pollinators [10]. Other physiological functions include defense against fungal pathogens and UV-B radiation [10]. They are also involved in energy transfer, growth regulation, respiration, photosynthesis, morphogenesis, and sex determination [10]. The basic structural feature of flavonoids is the 2-phenyl-benzo[α]pyrane or flavane nucleus, which consists of two benzene rings (A and B) linked through a heterocyclic pyrane ring (C) (Figure 2).

Flavonoid compounds may be classified based on their biosynthetic origins, as follows: aurone, isoflavone, chalcone, flavanone, flavone, flavonol, flavanon-3-ol, anthocyanidin, flavan-3-ol, and flavan. Various studies showed flavonoids exhibit antimicrobial properties; examples include apigenin, galangin, pinocembrin, ponciretin, genkwanin, sophoraflavanone G and its derivatives, naringenin, epigallocatechin gallate and its derivatives, luteolin, quercetin, 3-*O*-methylquercetin, and kaempferol and its derivatives [11]. Most of these compounds have low toxicity towards mammals, making them attractive templates for drug development.

Herein, we report the identification of various flavonoids as potential inhibitors for DHQase from *E. faecalis* (efDHQase). The study also elucidated the structure of *E. faecalis* DHQase to a resolution of 2.2 Å. This study provides significant biochemical and structural information that will facilitate the future development of polyketide-based antimicrobial inhibitors targeting the shikimate pathway of the nosocomial pathogen *E. faecalis*.

## Materials and Methods

### Cloning, expression and purification of 3-dehydroquinate dehydratase from *Enterococcus faecalis* (efDHQase)

The *aroD* gene encoding 3-dehydroquinate dehydratase (efDHQase, 3-dehydroquinate dehydratase from *Enterococcus faecalis* V583 strain) (GI: 29376281) was amplified *via* PCR from genomic DNA isolated from *E. faecalis* V583 strain using Platinum *Pfx* DNA polymerase (Invitrogen). The PCR mixture (100 µL) contained 1 ng of plasmid DNA, 10 µL of 10× Pfx amplifi cation buffer, 1 mM MgSO_4_, dNTPs (0.4 mM each), 40 pmol of each primer (forward primer 5**′**-GAGGAGGGCGCATATGAGCAACCATCTTTTCGACG-3**′** and reverse primer 5**′**-GAGAGCGCGGGATCCTTACGTCCTGGTATAAAGATC-3**′** ), and 5 units of Platinum *Pfx* DNA polymerase. The gene was amplified using a PTC-0200G Thermal Cycler (Bio-Rad Laboratories), with the following parameters: 94°C for 2 min followed by 40 cycles of 94°C for 1 min, 55°C for 1 min and 15 s, and 68°C for 3 min, and a final extension of 68°C for 10 min. The amplified gene was cloned into a modified pET-15b vector (Novagen) in which the N-terminus contained 10 His residues (kindly provided by Professor John Gerlt, University of Illinois, Urbana, IL) [12]. The protein was expressed in *E. coli aroD^−^ pTara*, an auxotroph *aroD^−^* negative *E. coli* mutant strain in which the *aroD* gene was deleted from the genome. Transformed cells were grown at 37°C in LB broth (supplemented with 100 µg/mL of ampicillin, 15 µg/mL of chloramphenicol and 50 µg/mL of kanamycin) to an OD_600_ of 0.6, and IPTG (0.1 mM) was added to induce protein expression for 16 h. The cells were harvested by centrifugation and resuspended in binding buffer [5 mM imidazole, 0.5 M NaCl, and 20 mM Tris-HCl (pH 7.9)] and lysed by sonication. The lysate was clarified by centrifugation, and the His-tagged protein was purified using a column of chelating Sepharose Fast Flow (GE Healthcare Bio-Sciences Corp.) charged with Ni^2+^ ion. The cell lysate was applied to the column in binding buffer, washed with buffer containing 154 mM imidazole, 0.5 M NaCl, and 20 mM Tris-HCl, pH 7.9, and eluted with 100 mM L-histidine, 0.5 M NaCl, and 20 mM Tris-HCl, pH 7.9. The N-terminal His tag was removed with thrombin (GE Healthcare Bio-Sciences Corp.) according to the manufacturer**'**s instructions, and the proteins were purified to homogeneity on a Q Sepharose High Performance column (GE Healthcare Bio-Sciences Corp.) equilibrated with binding buffer [25 mM Tris-HCl, pH 7.9] and eluted with a linear gradient from 0 to 0.5 M elution buffer [1 M NaCl and 25 mM Tris-HCl, pH 7.9].

### Cloning, expression and purification of shikimate dehydrogenase from *Enterococcus faecalis* (efSHD)

The *aroE* gene encoding shikimate dehydrogenase (efSHD) (GI: 29343586) was amplified *via* PCR from genomic DNA isolated from *E. faecalis* V583 strain using Platinum *Pfx* DNA polymerase (Invitrogen). The PCR mixture (100 µL) contained 1 ng of plasmid DNA, 10 µL of 10× Pfx amplification buffer, 1 mM MgSO_4_, dNTPs (0.4 mM each), 40 pmol of each primer (forward primer 5**′**-AAGGAGCACACCATATGAAAGAAATAACTGGAGCCACTCG-3**′** and reverse primer 5**′**-TAAACGGCGGGATCCTTATCTATTTTCAATTTTACGTTTTACG-3**′** ), and 5 units of Platinum *Pfx* DNA polymerase. The gene was amplified using a PTC-0200G Thermal Cycler (Bio-Rad Laboratories), with the following parameters: 94°C for 2 min followed by 40 cycles of 94°C for 1 min, 55°C for 1 min and 15 s, and 68°C for 3 min, and a final extension of 68°C for 10 min. The amplified gene was cloned into the modified pET-15b vector (Novagen) [12]. The protein was expressed in *E. coli aroE^−^ pTara*, an auxotroph *aroE^−^* negative *E. coli* mutant strain in which the *aroE* gene was deleted from the genome. Transformed cells were grown at 37°C in LB broth (supplemented with 100 µg/mL of ampicillin, 15 µg/mL of chloramphenicol and 50 µg/mL of kanamycin) to an OD_600_ of 0.6, and IPTG (0.1 mM) was added to induce protein expression for 16 h. efSHD was purified from the harvested cells as previously described for efDHQase.

### Crystallization and structure solution of efDHQase

Freshly prepared efDHQase was concentrated to 30 mg/mL. Crystals grew within 5 days at 25°C by the sitting-drop vapor diffusion method from a 1∶1 (v/v) mixture with precipitant solution comprised of 100 mM HEPES sodium salt, pH 7.5, 30% (w/v) PEG 4000, and 0.2 M calcium chloride. Crystals were soaked in the precipitant solution supplemented with 20% (v/v) glycerol, and flash frozen in liquid nitrogen. X-ray diffraction data were collected at 100 K on a Bruker X8 PROTEUM system consisting of a MICROSTAR micro-focus X-ray generator, a PLATINUM135 CCD detector, and a 4-circle KAPPA goniometer. Data reduction was carried out using SAINT, SADABS, and XPREP, within the Bruker Proteum2 program suite [13], [14].

Structural determination of efDHQase was initiated by molecular replacement using the structure of dehydroquinate dehydratase from *Clostridium difficile* (PDB ID: 3JS3, chain A) as the model. The model, together with the processed data and the amino acid sequence of efDHQas**e**, was fed into MrBUMP [15], an automated molecular replacement pipeline in the CCP4 suite [16]. CHAINSAW [17], PHASER [18] and REFMAC5 [19] were used within this pipeline to automatically trim the model, search for its correct rotation and translation, and refine it, respectively. The solution was then subjected to five rounds of automated model building using ARP/wARP [20], followed by restrained refinement in PHENIX [21] and manual building in COOT [22]. TLS refinement was included in the later rounds of PHENIX refinement. All the structure-related figures were prepared with the PyMOL Molecular Graphics System (DeLano Scientific LLC). Data collection and refinement statistics are listed in Table 1. Attempts at obtaining inhibitor-liganded structures of efDHQase were unsuccessful.

**Table 1:** Data Collection, Refinement and Structure Validation Statistics.

**Data collection**	
Space group	P1
Cell dimensions	
a, b, c (Å)	42.97, 48.29, 67.43
alpha, beta, gamma (°)	108.07, 92.86, 111.83
Resolution (Å)	19.8 – 2.2 (2.3 – 2.2)
Rmerge (%)	5.9 (20.7)
I/sigma(I)	9.2 (3.7)
Completeness (%)	89.2 (49.0)
Redundancy	3.4 (1.4)
**Refinement**	
Resolution (Å)	19.8 – 2.2 (2.3- 2.2)
No. reflections	21267 (1063)
R_work_/R_free_ (%)	18.5 (25.5)/18.2 (24.2)
No. atoms	
Protein	3746
Water	311
*B*-factors	
Protein	15.6
Water	22.3
R.m.s. deviations	
Bond lengths (Å)	0.008
Bond angles (°)	1.042
**MOLPROBITY validation**	
Poor rotamers	0.97% (Goal: <1%)
Ramachandran outliers	0% (Goal: <0.2%)
Ramachandran favored	98.9% (Goal: >98%)
Cβ deviations >0.25 Å	0 (Goal: 0)
Residues with bad bonds:	0% (Goal: 0%)
Residues with bad angles:	0% (Goal: <0.1%)

### Kinetic parameters of efDHQase

**Figure 3:**
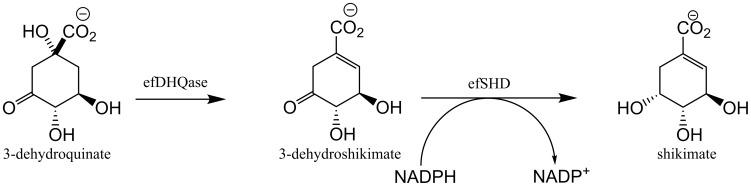
efDHQase-efSHD coupled-enzyme assay.

The activity of efDHQase was measured using a continuous coupled-enzyme spectrophotometric assay monitored at 25°C. The rate of dehydroshikimate formation was coupled to the oxidation of NADPH (340 nm, ε = 6220 M^−1^ cm^−1^) (Figure 3). The 200 µL assay contained 50 mM HEPES buffer, pH 7.5, 0.25 mM NADPH, 0.25 mM 3-dehydroquinate, 10 nM DEF and 10 nM shikimate dehydrogenase (efSHD). The kinetic parameters were determined by fitting the initial rates to the Michaelis-Menten equation.

### Screening for inhibitors targeting DEF

A library of 139 commercially available compounds containing different families of flavonoids was used for the screening. They were purchased from Sigma-Aldrich Co. (St. Louis, MO), Tokyo Chemical Industry (TCI) Co., Extrasynthese Co., and Lier Chemical Co. (Sichuan, China). The compounds are acacetin, aesculine, apigenin, apigenin-7-glucoside, apigeninidin chloride, apiin, biochanin, butein, butin, D-catechin tetrahydrate, (−)-catechin, (+)-catechin, (±)-catechin, chalcone, chrysin, cinnamic acid, coumarin, cyanidanol-3, cyaniding chloride, cyanin chloride, daidzein, daidzin, daphnetin, datiscetin, delphinidin chloride, dihydrofisetin, dihydroquercetin, dihydrorobinetin, 2,6-dihydroxyacetophenone, 2,3-dihydroxybenzoic acid, 2,4-dihydroxybenzoic acid, 3,4-dihydroxyhydrocinnamic acid, 4′,7-dihydroxyflavanol, 5,7-dihydroxy-4′-methoxyflavone, 5,7-dihydroxy-3′,4′,5′-trimethoxyflavone, 2,4-dimethoxycinnamic acid, 3,4-dimethoxycinnamic acid, 3′,4′-dihydroxyflavone, 2′,6′-dihydro-4′-methoxydihydrochalcone, diosmetin, diosmin, (−)-epicatechin, (−)-epicatechin gallate, (−)-epicatechol, (±)-epicatechol, (−)-epigallocatechin, epigallocatechin gallate, eriodictyol, eriodictyol-7-glucoside, ferrulic acid, ferulic acid, fisetin, (−)-fisetinidol, fisetinidin chloride, flavanone, flavanone hydrazone, flavanone-azine, flavene, flavone, flavonol, flavonone, formononetin, fustin, galangin, gallic acid, genistein, genistin, gossypin, hamamelitannin, hesperetin, hesperidin, homoorientin, 2-hydroxychalcone, 4-hydroxychalcone, 4-hydroxycinnamic acid, 3-hydroxycoumarin, 4-hydroxycoumarin, 4′-hydroxyflavanone, 3-hydroxyflavone, 5-hydroxyflavone, 6-hydroxyflavone, 7-hydroxyflavone, 5-hydroxyflavonone, *p*-hydroxyphenylacetic acid, isoquercitrin, isorhamnetin, kaempferol, kinetin, leucocyanidin hydrate, linarin, luteolin, luteolin-3′,7-Diglucoside, luteolin-4′-glucoside, luteolin-7-glucoside, luteolinidin chloride, marein, maritimein, 2-methoxycinnamic acid, 4-methoxycinnamic acid, methylchalcone, α-methylcinnamic acid, 4-methylcinnamic acid, β-methylumbelliferone, mono-7-rutin, morin, myricetin, myricitrin, naringenin, naringin, orientin, phenylacetic acid, 2-phenylchromone, phloretin, phloridzin, phloridzin dihydrate, puerarin, quebrachitol, quercetin, quercitrin, quercitrine, rhamnetin, rhoifolin, robinetin, robinin, rutin, rutinose, sakuranetin, scopoletin, (−)-shikimic acid, silybin, sinapic acid, sulfuretin, tangeretin, (+)-taxifolin, techtochrysin, 3,5,7-trihydroxyflavone, 4′,6,7-trihydroxyisoflavone, umbelliferone, and veratric acid, respectively.

The efDHQase coupled-enzyme assay was used to identify potential inhibitors from the library. Flavonoids that decreased the rate of dehydroshikimate formation by at least 50% were considered as potential efDHQase or efSHD inhibitors.

**Figure 4:**
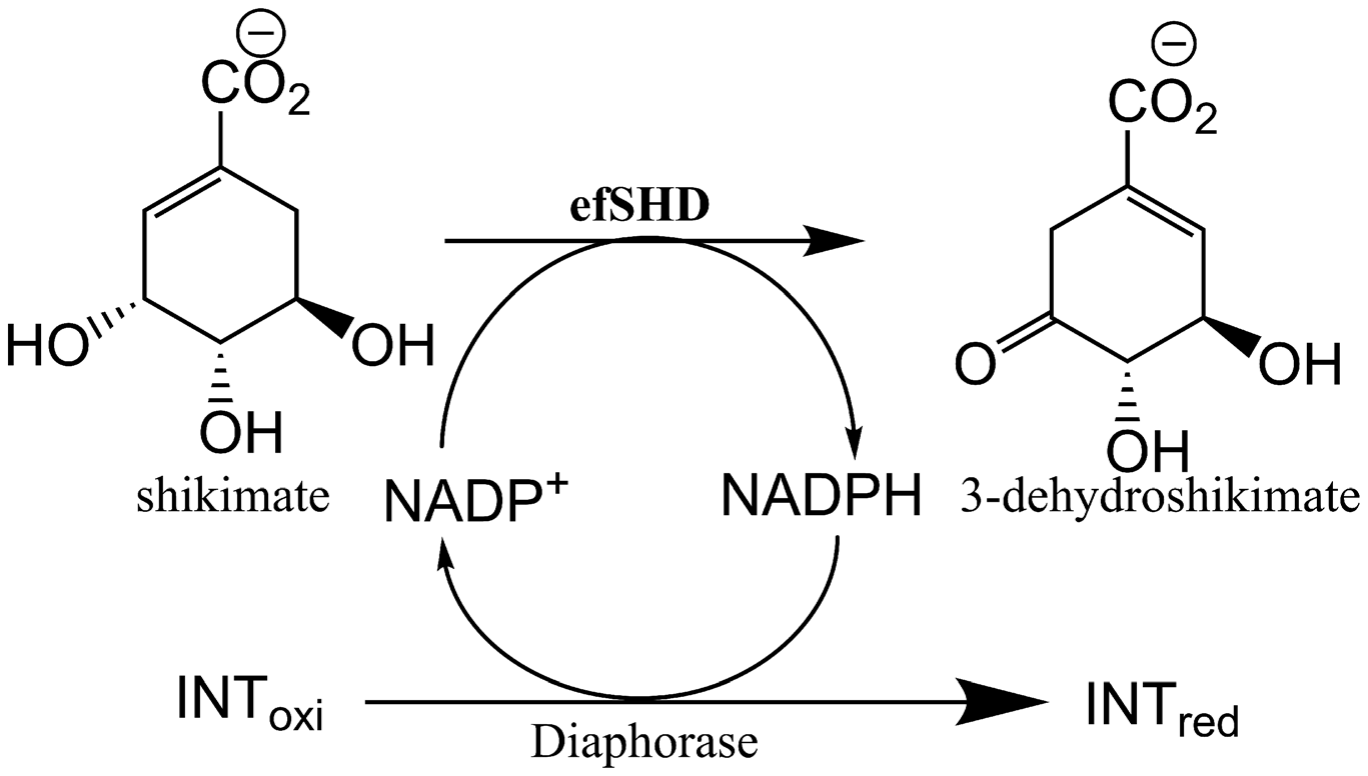
efSHD-diaphorase coupled-enzyme assay.

Flavonoids that inhibited the efDHQase coupled-enzyme assay may inhibit efSHD instead of efDHQase. Thus, efSHD was assayed independently. efSHD was assayed using a continuous coupled-enzyme spectrophotometric assay (Figure 4) monitored at 25°C. The rate of 3-dehydroshikimate formation was coupled to the reduction of *p*-iodonitrotetrazolium (INT) (500 nm, ε = 17800 M^−1^ cm^−1^). The 200 µL assay contained 50 mM HEPES buffer, pH 7.5, 0.15 mM NADP^+^, 1.5 mM shikimate, 5 units of diaphorase (Sigma), 1 mM INT, and 10 nM efSHD. Flavonoids that did not affect the rate of dehydroshikimate formation were considered as potential efDHQase inhibitors.

### Determination of minimal inhibition concentration against *E. faecalis*

A minimal inhibition concentration (MIC) assay using the flavonoid library was performed to screen for compounds targeting the growth of *E. faecalis*. The assay was based on the standard guidelines from the National Committee for Clinical Laboratory Standards (NCCLS), sixth edition. Briefly, turbidity-adjusted bacterial suspensions from fresh overnight cultures were added to LB broth to conform to an inoculation density of 5×10^5^ cfu/mL. Each 100 µL bacterial culture contained 2 mM flavonoid and was incubated for 17 h at 37°C. After incubation, the cell density at OD_600_ was measured for each culture. If the cell density of the flavonoid containing culture is less than 50% compared to the cultures that did not have any flavonoid, these flavonoids were chosen as potential inhibitors.

Flavonoids used at 2 mM that inhibited *E. faecalis* growth by at least 50% were further tested. The concentration of each flavonoid was continually decreased until the lowest concentration of the compound that inhibited *E. faecalis* growth by at least 50% was obtained. This concentration is calculated to be the MIC for each flavonoid.

## Results and Discussion

### Crystal structure of efDHQase

**Figure 5:**
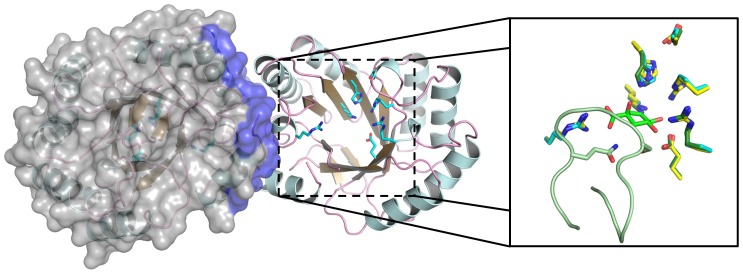
Crystal structure of efDHQase. The biological assembly of efDHQase is a homodimer. Alpha-helices, β-strands, and loops are shown as cartoon representations and colored light blue, golden, and pink, respectively, to visualize the (β/α)_8_-barrel scaffold on each monomer. Residues at the dimer interface are highlighted purple on the surface (grey) of one monomer. Residues involved in ligand binding are shown as sticks and colored cyan. Comparison of efDHQase's active center with homologous 3-dehydroquinate dehydratases from *Salmonella enterica* (seDHQase) and *Clostridium difficile* (cdDHQase) is shown in the magnified view on the right. The structures were superimposed onto each other using the Cα atoms of the ligand-binding residues, with the residues from seDHQase and cdDHQase colored dark green and yellow, respectively. The pre-dehydration intermediate covalently-attached to seDHQase is drawn as green sticks. Part of the β8-α8 loop from seDHQase is shown as light green cartoon representations.

The crystal structure of efDHQase was solved using the Protein Crystallography X-Ray Facility hosted by the Institute of Molecular and Cell Biology, A*STAR Singapore. The efDHQase monomer has a (β/α)_8_-barrel scaffold (Figure 5). The majority of the molecule is well ordered and with clear density in the electron density map, with the exceptions of the first and last two residues at the N- and C-terminus, respectively, and a disordered portion (Ala228-Gln236) within the loop connecting the 8th β-strand and the 8th α-helix (β8-α8 loop). There are two monomers in the asymmetric unit of the crystal, forming a homodimer. Using the Protein Interfaces, Surfaces and Assemblies (PISA) service at the European Bioinformatics Institute (https://www.ebi.ac.uk/msd-srv/prot_int/cgi-bin/piserver) [23], the interface area between the two monomers (colored purple in Figure 5) was calculated to be 990.5 Å, or about 9% of one monomer's solvent-accessible surface area. On each monomer, there are a total of 94 atoms belonging to 24 residues that are involved in the formation of the dimer interface. The non-crystallographic symmetry (NCS) differences for most of the residues are well below 0.3 Å (as reported by COOT [22]), indicating close conformational similarity between the two protomers.

To gain further insight into efDHQase's enzymatic activity, we compared the efDHQase structure with homologous DHQase structures from *Salmonella enterica* (seDHQase) and *Clostridium difficile* (cdDHQase). These homologous enzymes share about 46% sequence identity with efDHQase, and have been characterized structurally for their ligand binding and Schiff base formation. The magnified view in Figure 5 shows the overlaid active centers of efDHQase and its homologs, seDHQase (PDB ID: 3M7W) and cdDHQase (PDB ID: 3JS3), that have been crystallized in the presence of covalently-bound pre- and post-dehydration intermediates, respectively [24]. Key residues, which are responsible for ligand coordination in the homologous structures, are well conserved in efDHQase (sticks representation). Moreover, these residues, and in particular Lys170, are positioned in nearly identically positions in these three homologous structures, despite the absence of bound ligand in the efDHQase structure. Such similarities strongly suggest that efDHQase catalyzes the dehydration reaction *via* a Schiff base mechanism whereby the terminal amine of efDHQase's Lys170 reversibly reacts with the carbonyl of the substrate [24], [25], [26], [27]. To reinforce this notion, the pre-dehydration covalent intermediate bound to seDHQase is also shown in Figure 5 (green sticks), with its carbonyl carbon in close proximity to efDHQase's Lys170. Among the key ligand-binding residues of the three overlaid structures, the side chain of His143 exhibits the largest conformational deviations, which is in line with the hypothesis that this residue utilizes its conformational flexibility to exert dual catalytic roles [24]. In the absence of bound ligand, the β8-α8 loop of efDHQase adopts the “open” conformation commonly seen in other DHQases [26], [27], and becomes partially disordered. To model the “closed” conformation of the β8-α8 loop, the undefined portion of the loop was taken from seDHQase and is shown in Figure 5 (light green). Since the residues in this portion are highly conserved among DHQases [27], the model is likely to have good credibility.

### Kinetic parameters of efDHQase

The turnover number (*k*_cat_) of efDHQase is 110 s^−1^ and the Michaelis constant (*K*_M_) for 3-dehydroquinate is 65 µM. This is comparable to DHQases from other type I DHQases such as *Escherichia coli* [28], *Salmonella typhi* [7], and *Streptococcus pneumoniae* [29] characterized under similar conditions (Table 2).

**Table 2:** Kinetic parameters of various DHQases.

Organism	k_cat_ (s^−1^)	K_M_ (µM)	k_cat_/K_M_ (M^−1^ s^−1^)
*Enterococcus faecalis*	110	65	1.7×10^6^
*Escherichia coli* [13]	135	18	7.5×10^6^
*Salmonella typhi* [7]	200	18	1.1×10^7^
*Streptococcus pneumoniae* [14]	32	74	4.3×10^5^

### Flavonoid inhibitors that inhibit efDHQase and/or *E. faecalis growth*

**Figure 6:**
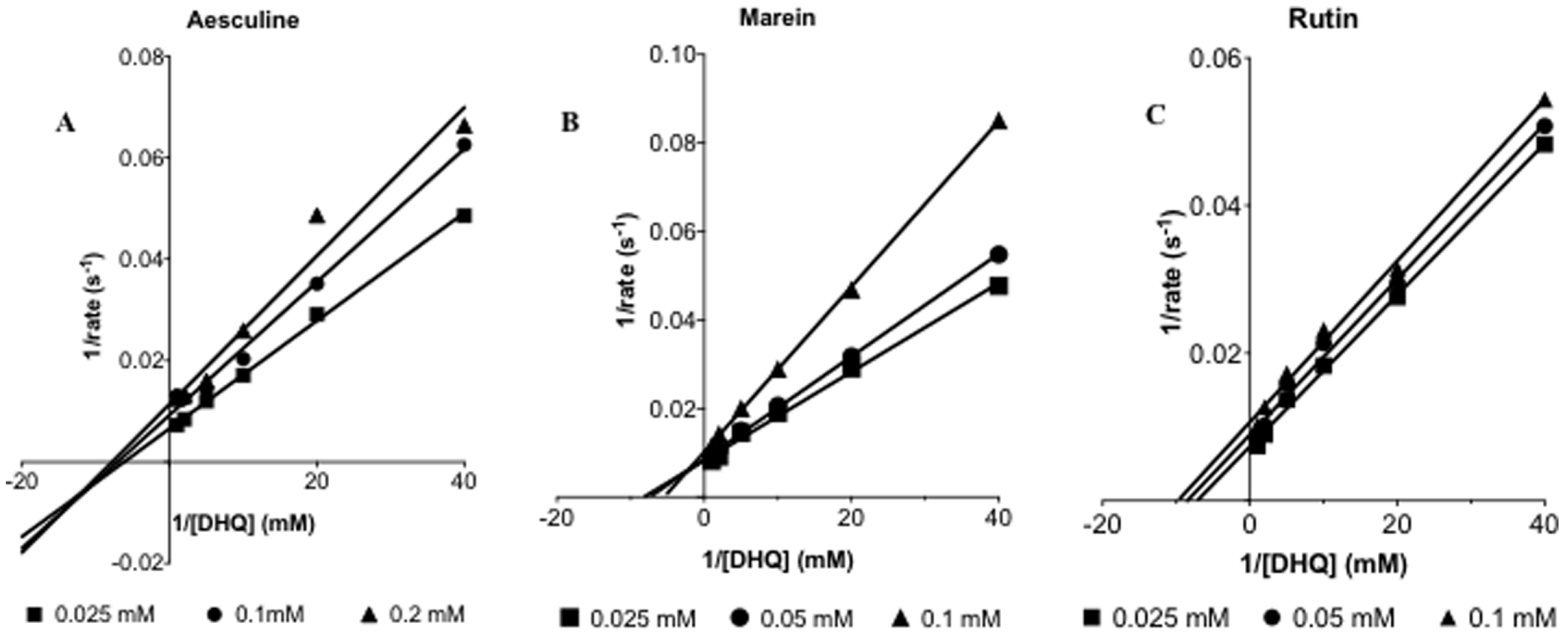
Double-reciprocal plots for three of the identified inhibitors targeting efDHQase. Plot for aesculine (A) and marein (B) showing two possible scenarios for mixed inhibition where the lines intersect either below the x-axis or just after y-axis respectively. Plot for rutin (C) showing uncompetitive inhibition where the lines run parallel to one another.

From a library of 139 commercially available flavonoid compounds, nine efDHQase inhibitors were found (Table 3). The flavonoid inhibitors exhibited either mixed or uncompetitive inhibition. Representative double-reciprocal plots to determine the type of inhibition are shown in Figure 6. The *K*_I_'s that were determined ranged from 20–350 µM, which is in the range of 3-dehydroquinate's Michaelis constant (65 µM).

**Table 3:** efDHQase inhibition constants (*K*_I_).

Compound	Type of flavonoid	*K*_I_ (µM)	Mode of inhibition
Aesculine	Coumarin glycoside	180±38	Mixed
Gossypin	Flavonoid glycoside	79±14	Mixed
Homoorientin	Flavonoid glycoside	140±11	Uncompetitive
7-Hydroxycoumarin	Coumarin	42±19	Uncompetitive
Luteolin -7-glucoside	Flavonoid glycoside	69±16	Mixed
Luteolin-3′-7-diglucoside	Flavonoid glycoside	89±3	Mixed
Marein	Chalconoid glycoside	340±79	Mixed
Rhoifolin	Flavonoid glycoside	19±9	Uncompetitive
Rutin	Flavonoid glycoside	210±28	Uncompetitive

Compounds that showed mixed inhibition are usually a mixture of competitive and non-competitive inhibition (Figure 6A and 6B, respectively). We propose that these compounds bind to either the enzyme or enzyme-substrate complex but with higher affinity for the former [30]. Some flavonoids showed uncompetitive inhibition (Figure 6C). We propose that these inhibitors only bind to the enzyme-substrate complex and delay the time for the substrate and product to leave the active site, thus causing a decrease in *V*_max_ [30].

Four compounds were identified as potential inhibitors with moderate MIC values, ranging from 500–1000 µM (Table 4).

**Table 4:** List of flavonoids inhibiting *Enterococcus faecalis*.

Compound	Type of flavonoid	MIC values (µg/mL)
Datiscetin	Flavone	256
Marein	Chalcone glycoside	256
Naringenin	Flavanone	512
Phloretin	Chalcone	512

The 139-compound library may be classified into flavonoids and its derivatives (anthocyanidin, chalcone, flavan, flavone, flavanone, isoflavone), aromatic acids (benzoic acids and cinnamic acids), and coumarins. Some of the flavonoids and coumarins are glycosides, with one or two sugar units. The compounds were chosen based on previous antimicrobial studies [11], [31], [32]

From this library, six flavonoid glycosides inhibited efDHQase while two flavonoids inhibited the growth of *E. facaelis*. The six flavonoid glycosides can be classified further based on their aglycone – a) gossypin and rutin are quercetin glycosides; b) homoorientin, luteolin-3′,7-diglucoside, and luteolin-7-glucoside are luteolin glycosides; and, c) rhoifolin is an apigenin glucoside. None of the six flavonoid glycosides inhibited the growth of *E. facaelis*. Two flavonoids, datiscetin and naringenin, inhibited the growth of *E. faecalis*. Datiscetin, a flavonol from *Datisca cannabina* (hemp), was previously found to inhibit the growth of methicillin-resistant *S. aureus* (MRSA) (MIC = 512 µg/mL) [33]. Naringenin, in combination with other flavonoids extracted from *Centaurea alexanderina*, was found to inhibit the growth of *P. aeruginosa* [34].

These observations are consistent with previous studies that found flavonoids inhibited bacterial growth but not their corresponding glycosides. Rauha found that quercetin inhibited the growth of Gram-positive bacteria such as *Bacillus subtilis, Micrococcus luteus, Staphylococcus aureus* and *S. epidermidis*, but not quercetin glycosides such as rutin [35]. Funayama also showed that flavonoids without the sugar moiety have antimicrobial activity on *B. subtilis*, *S. aureus*, and *M. luteus*, but their corresponding glycosides did not [32]. Pereira found that a combination of flavonoid glycosides is needed to inhibit bacterial growth; extracts containing luteolin-7-glucoside, rutin, apigenin-7-glucoside and luteolin-4′-glucoside from *Olea europaea L.* (olive tree) were found to inhibit a range of Gram-positive bacteria such as *B. cereus*, *B. subtilis*, and *S. aureus* [36].

**Figure 7:**
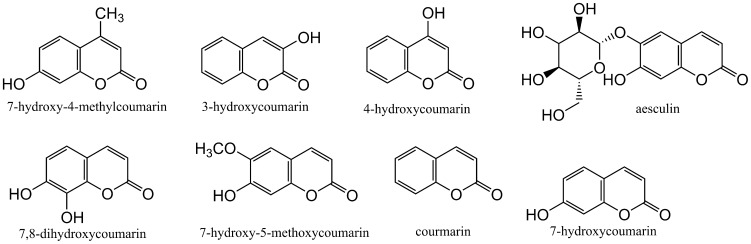
Coumarins in the 139-compound polyketide-based library.

Coumarins are a large class of phenolic substances made of fused benzene and α-pyrone rings, which constitute the core skeleton of many flavonoid compounds. The library contained eight coumarins (Figure 7) but only 7-hydroxycoumarin and aesculin showed significant inhibition against efDHQase. It is possible that small perturbations to the coumarin structure greatly affected the binding to efDHQase. Both compounds did not inhibit the growth of *E. facaelis*. 7-Hydroxycoumarin was previously found to have antimicrobial properties against *Paecilomyces fulva*, a plant pathogen that causes Byssochlamys rot on strawberries [37]. Aesculin was previously shown not to have antimicrobial activity against *S. aureus, E.coli, and P. aeruginosa* [38].

A review by Venugopala postulated that long chain hydrocarbon substitutions on coumarins are needed for antibacterial activity [39]. Compounds such as ammoresinol and ostruthin showed activity against Gram-positive bacteria such as *B. megaterium*, *M. luteus*, *M. lysodeikticus*, and *S. aureus* [40]. Anthogenol, from green fruits of *Aegle marmelos*, showed activity against *Enterococcus* [39]. Chartreusin and its derivatives were found to be active against methicillin-resistant *S. aureus* (MRSA), vancomycin-resistant *Enterococcus* (VRE), and *Mycobacterium vaccae* but these compounds are highly toxic [41].

Chalcones are open-chain flavonoids with α,β-unsaturated carbonyl groups and is one of the important compound groups of flavonoids derived from nature. Out of the eight chalcones in the library, two of them, phloretin and marein, inhibited the growth of *E. facaelis* (MIC≤512 µg/mL). Phloretin, a chalcone found in leaves of *Malus domestica* (apple), is known to have antitumor and antioxidant properties. A study by Osório found that it inhibited fourteen strains of MRSA (MIC = 250 µg/mL) [42].

Marein is the only compound that inhibited both efDHQase activity and *E. facaelis* growth. It is a chalcone glucoside found on *Coreopsis maritime*, functioning as one of the ***anthochlor*** pigments of this flower. It was recently found that it inhibited histone deacetylase enzymes and NF-κB, a protein complex that controls the transcription of DNA, in the 100 µM range [43]. It was also observed to have antihyperglycemic activity [44].

Various studies have shown that although chalcones weakly inhibits bacterial growth, adding conventional antibiotics greatly increases their antimicrobial activity [45]. A study by Tran observed better antibacterial activity when a combination of chalcones and antibiotics such as ciprofloxacin and doxycycline were used [46].

### Summary

This study has demonstrated the utilization of flavonoids as potential drug leads. We have observed inhibition against efDHQase activity and *E. facaelis* growth using flavonoids and its glycosides, chalcones, and coumarins. From a 139-compound polyketide-based library, we found that flavonoids inhibited growth while its glycosides inhibited crucial metabolic enzymes such as efDHQase. In addition, we observed that small perturbations in the coumarin structure greatly affected the ability of the polyketide-based small molecule compounds to bind to the enzyme. Lastly, chalcones inhibited bacterial growth but a synergy with more conventional antibiotics may be needed for exerting potent inhibition. We have also elucidated the structure of efDHQase. Our work provides a route in the development of polyketide-based antimicrobial inhibitors targeting the shikimate pathway of the human pathogen *E. faecalis*.

## References

[1] Herrmann KM, Weaver LM (1999) The Shikimate Pathway. Annu Rev Plant Physiol Plant Mol Biol 50: 473–503. 15012217 10.1146/annurev.arplant.50.1.473

[2] Coggins JR, Abell C, Evans LB, Frederickson M, Robinson DA, et al (2003) Experiences with the shikimate-pathway enzymes as targets for rational drug design. Biochem Soc Trans 31: 548–552. 12773154 10.1042/bst0310548

[3] Hollander H, Amrhein N (1980) The Site of the Inhibition of the Shikimate Pathway by Glyphosate: I. Inhibition by glyposphate of phenylpropanoid synthesis in buckwheat (*Fagopyrum esculentum Moench*). Plant Physiol 66: 823–829. 16661534 10.1104/pp.66.5.823 PMC440734

[4] Jaworski EG (1972) Mode of action of N-phosphonomethylglycine. Inhibition of aromatic amino acid biosynthsis. Journal of Agricultural and Food Chemistry 20: 1195–1198.

[5] Steinrucken HC, Amrhein N (1980) The herbicide glyphosate is a potent inhibitor of 5-enolpyruvyl-shikimic acid-3-phosphate synthase. Biochem Biophys Res Commun 94: 1207–1212. 7396959 10.1016/0006-291x(80)90547-1

[6] Chaudhuri S, Lambert JM, McColl LA, Coggins JR (1986) Purification and characterization of 3-dehydroquinase from *Escherichia coli* . Biochem J 239: 699–704. 2950851 10.1042/bj2390699 PMC1147342

[7] Moore JD, Hawkins AR, Charles IG, Deka R, Coggins JR, et al (1993) Characterization of the type I dehydroquinase from *Salmonella typhi* . Biochem J 295 (Pt 1) 277–285. 8216229 10.1042/bj2950277 PMC1134850

[8] Walker JC, Verma NK (1997) Cloning and characterisation of the aroA and aroD genes of *Shigella dysenteriae* type 1. Microbiol Immunol 41: 809–813. 9403507 10.1111/j.1348-0421.1997.tb01932.x

[9] Agrawal AD (2011) Pharmacological activities of flavonoids: a review. Int J Pharm Sci Nanotechnol 4: 1394–1398 1395 pp.

[10] Tomas-Barberan FA (2001) The Handbook of Natural Flavonoids, 2 volume set, Edited by J. B. Harborne and H. Baxter. Phytochem Anal 12: 214.

[11] Cushnie TP, Lamb AJ (2005) Antimicrobial activity of flavonoids. Int J Antimicrob Agents 26: 343–356. 16323269 10.1016/j.ijantimicag.2005.09.002 PMC7127073

[12] Yew WS, Gerlt JA (2002) Utilization of L-ascorbate by *Escherichia coli* K-12: assignments of functions to products of the yjf-sga and yia-sgb operons. J Bacteriol 184: 302–306. 11741871 10.1128/JB.184.1.302-306.2002 PMC134747

[13] Bruker AXS (2010) PROTEUM2. Bruker AXS Inc., Madison, Wisconsin, USA.

[14] Sheldrick GM (2008) A short history of SHELX. Acta crystallographica Section A, Foundations of crystallography 64: 112–122. 18156677 10.1107/S0108767307043930

[15] Keegan RM, Winn MD (2007) Automated search-model discovery and preparation for structure solution by molecular replacement. Acta Crystallographica Section D Biological Crystallography 63: 447–457. 17372348 10.1107/S0907444907002661

[16] Winn MD, Ballard CC, Cowtan KD, Dodson EJ, Emsley P, et al (2011) Overview of the CCP4 suite and current developments. Acta crystallographica Section D, Biological crystallography 67: 235–242. 21460441 10.1107/S0907444910045749 PMC3069738

[17] Stein N (2008) CHAINSAW: a program for mutating pdb files used as templates in molecular replacement. Journal of Applied Crystallography 41: 641–643.

[18] McCoy AJ, Grosse-Kunstleve RW, Adams PD, Winn MD, Storoni LC, et al (2007) Phaser crystallographic software. J Appl Crystallogr 40: 658–674. 19461840 10.1107/S0021889807021206 PMC2483472

[19] Murshudov GN, Skubák P, Lebedev AA, Pannu NS, Steiner RA, et al (2011) REFMAC5 for the refinement of macromolecular crystal structures. Acta crystallographica Section D, Biological crystallography 67: 355–367. 21460454 10.1107/S0907444911001314 PMC3069751

[20] Langer G, Cohen SX, Lamzin VS, Perrakis A (2008) Automated macromolecular model building for X-ray crystallography using ARP/wARP version 7. Nature protocols 3: 1171–1179. 18600222 10.1038/nprot.2008.91 PMC2582149

[21] Adams PD, Afonine PV, Bunkoczi G, Chen VB, Davis IW, et al (2010) PHENIX: a comprehensive Python-based system for macromolecular structure solution. Acta Crystallogr D Biol Crystallogr 66: 213–221. 20124702 10.1107/S0907444909052925 PMC2815670

[22] Emsley P, Lohkamp B, Scott WG, Cowtan K (2010) Features and development of Coot. Acta Crystallogr D Biol Crystallogr 66: 486–501. 20383002 10.1107/S0907444910007493 PMC2852313

[23] Krissinel E, Henrick K (2007) Inference of macromolecular assemblies from crystalline state. J Mol Biol 372: 774–797. 17681537 10.1016/j.jmb.2007.05.022

[24] Light SH, Minasov G, Shuvalova L, Duban M-E, Caffrey M, et al (2011) Insights into the mechanism of type I dehydroquinate dehydratases from structures of reaction intermediates. The Journal of biological chemistry 286: 3531–3539. 21087925 10.1074/jbc.M110.192831 PMC3030358

[25] Gourley DG, Shrive AK, Polikarpov I, Krell T, Coggins JR, et al (1999) The two types of 3-dehydroquinase have distinct structures but catalyze the same overall reaction. Nature structural biology 6: 521–525. 10360352 10.1038/9287

[26] Light SH, Antanasijevic A, Krishna SN, Caffrey M, Anderson WF, et al (2014) Crystal structures of type I dehydroquinate dehydratase in complex with quinate and shikimate suggest a novel mechanism of schiff base formation. Biochemistry 53: 872–880. 24437575 10.1021/bi4015506 PMC3985847

[27] Light SH, Minasov G, Shuvalova L, Peterson SN, Caffrey M, et al (2011) A conserved surface loop in type I dehydroquinate dehydratases positions an active site arginine and functions in substrate binding. Biochemistry 50: 2357–2363. 21291284 10.1021/bi102020s PMC3062685

[28] Chaudhuri S, Duncan K, Graham LD, Coggins JR (1991) Identification of the active-site lysine residues of two biosynthetic 3-dehydroquinases. Biochem J 275 (Pt 1) 1–6. 1826831 10.1042/bj2750001 PMC1150004

[29] Noble M, Sinha Y, Kolupaev A, Demin O, Earnshaw D, et al (2006) The kinetic model of the shikimate pathway as a tool to optimize enzyme assays for high-throughput screening. Biotechnol Bioeng 95: 560–573. 16921527 10.1002/bit.20772

[30] Segel IH (1975) Enzyme Kinetics: Behavior and Analysis of Rapid Equilibrium and Steady-State Enzyme Systems: Wiley. 982 pp. p.

[31] Cowan MM (1999) Plant products as antimicrobial agents. Clin Microbiol Rev 12: 564–582. 10515903 10.1128/cmr.12.4.564 PMC88925

[32] Funayama S, Komiyama K, Miyaichi Y, Tomimori T, Nozoe S (1995) Cytocidal and antimicrobial activities of flavonoids. Nat Med 49: 322–328.

[33] Xu H-X, Lee SF (2001) Activity of plant flavonoids against antibiotic-resistant bacteria. Phytotherapy Research 15: 39–43. 11180521 10.1002/1099-1573(200102)15:1<39::aid-ptr684>3.0.co;2-r

[34] Kubacey TM, Haggag EG, El-Toumy SA, Ahmed AA, El-Ashmawy IM, et al. (2012) Biological activity and flavonoids from Centaurea alexanderina leaf extract.

[35] Rauha JP, Remes S, Heinonen M, Hopia A, Kahkonen M, et al (2000) Antimicrobial effects of Finnish plant extracts containing flavonoids and other phenolic compounds. Int J Food Microbiol 56: 3–12. 10857921 10.1016/s0168-1605(00)00218-x

[36] Pereira AP, Ferreira ICFR, Marcelino F, Valentao P, Andrade PB, et al (2007) Phenolic compounds and antimicrobial activity of olive (*Olea europaea L. cv. cobrancosa*) leaves. Molecules 12: 1153–1162. 17873849 10.3390/12051153 PMC6149345

[37] Jurd L, King AD Jr, Mihara K (1971) Antimicrobial properties of umbelliferone derivatives. Phytochemistry 10: 2965–2970.

[38] Kostova IN, Nikolov NM, Chipilska LN (1993) Antimicrobial properties of some hydroxycoumarins and *Fraxinus ornus* bark extracts. Journal of Ethnopharmacology 39: 205–208. 8258978 10.1016/0378-8741(93)90037-6

[39] Venugopala KN, Rashmi V, Odhav B (2013) Review on natural coumarin lead compounds for their pharmacological activity. BioMed Res Int 963248, 963214. 23586066 10.1155/2013/963248 PMC3622347

[40] Hodak K, Jakesova V, Dadak V (1967) On the antibiotic effects of natural coumarins. VI. The relation of structure to the antibacterial effects of some natural coumarins and the neutralization of such effects. Cesk Farm 16: 86–91. 6044315

[41] Ueberschaar N, Xu Z, Scherlach K, Metsä-Ketelä M, Bretschneider T, et al (2013) Synthetic Remodeling of the Chartreusin Pathway to Tune Antiproliferative and Antibacterial Activities. Journal of the American Chemical Society 135: 17408–17416. 24143864 10.1021/ja4080024

[42] Moreira Osório T, Delle Monache F, Domeneghini Chiaradia L, Mascarello A, Regina Stumpf T, et al (2012) Antibacterial activity of chalcones, hydrazones and oxadiazoles against methicillin-resistant Staphylococcus aureus. Bioorg Med Chem Lett 22: 225–230. 22169259 10.1016/j.bmcl.2011.11.059

[43] Orlikova B, Schnekenburger M, Zloh M, Golais F, Diederich M, et al (2012) Natural chalcones as dual inhibitors of HDACs and NF-kappaB. Oncol Rep 28: 797–805. 22710558 10.3892/or.2012.1870 PMC3583578

[44] Dias T, Liu B, Jones P, Houghton PJ, Mota-Filipe H, et al (2012) Cytoprotective effect of Coreopsis tinctoria extracts and flavonoids on tBHP and cytokine-induced cell injury in pancreatic MIN6 cells. J Ethnopharmacol 139: 485–492. 22143153 10.1016/j.jep.2011.11.038

[45] Eumkeb G, Siriwong S, Phitaktim S, Rojtinnakorn N, Sakdarat S (2012) Synergistic activity and mode of action of flavonoids isolated from smaller galangal and amoxicillin combinations against amoxicillin-resistant Escherichia coli. J Appl Microbiol 112: 55–64. 22111967 10.1111/j.1365-2672.2011.05190.x

[46] Tran T-D, Do T-H, Tran N-C, Ngo T-D, Huynh T-N-P, et al (2012) Synthesis and anti Methicillin resistant Staphylococcus aureus activity of substituted chalcones alone and in combination with non-beta-lactam antibiotics. Bioorg Med Chem Lett 22: 4555–4560. 22727643 10.1016/j.bmcl.2012.05.112

